# Visceral Adipose Tissue Assessment Enhances the Prognostic Value of GLIM Criteria in Patients with Gastric Cancer Undergoing Radical Gastrectomy after Neoadjuvant Treatment

**DOI:** 10.3390/nu14235047

**Published:** 2022-11-27

**Authors:** Yingjing Zhang, Lin Jiang, Pengfei Su, Tian Yu, Zhiqiang Ma, Weiming Kang, Yuqin Liu, Zhengyu Jin, Jianchun Yu

**Affiliations:** 1 Department of General Surgery, Peking Union Medical College Hospital, Chinese Academy of Medical Sciences and Peking Union Medical College, Beijing 100730, China; 2 Department of Pathology, Institute of Basic Medical Sciences, Chinese Academy of Medical Sciences and Peking Union Medical College, Beijing 100730, China; 3 Department of Radiology, Peking Union Medical College Hospital, Chinese Academy of Medical Sciences and Peking Union Medical College, Beijing 100730, China

**Keywords:** GLIM, malnutrition, visceral adipose tissue, gastric cancer, clinical outcomes

## Abstract

Background: The Global Leadership Initiative on Malnutrition (GLIM) criteria has been recently published for diagnosing malnutrition in adults. However, the validity of the GLIM criteria has not been well-established in patients with gastric cancer (GC) treated with neoadjuvant treatment (NT) followed by radical gastrectomy. The present study aimed to explore the prognostic value of GLIM-defined malnutrition before NT and after NT in GC patients and to investigate whether additional visceral adipose tissue (VAT) assessment could improve the predictive power of the GLIM criteria for NT-related adverse events (AEs) and long-term survival. Methods: GC patients who underwent radical surgery after NT from June 2016 to June 2020 were enrolled in this study. The cross-sectional areas of total skeletal muscle (TSM) and VAT were measured using computed tomography (CT) before NT and after NT. GLIM-defined malnutrition was diagnosed using the two-step approach, including nutritional risk screening and diagnostic assessment. Low VAT was also added to the diagnosis of malnutrition in this study. The predictive value of these malnutrition diagnoses for NT-related AEs, and long-term survival was evaluated in GC patients. Results: A total of 182 GC patients were included in this study, of which 66 (36.3%) patients before NT and 55 (30.2%) patients after NT were diagnosed with GLIM-defined malnutrition, respectively. In addition to GLIM-defined malnutrition, 54 (29.7%) patients had additional low VAT before NT, and 39 (21.4%) patients had additional low VAT after NT. GLIM-defined malnutrition alone before NT was not associated with NT-related AEs in GC patients. The addition of low VAT to GLIM-defined malnutrition led to a significant predictive value for NT-related AEs. Furthermore, GLIM-defined malnutrition before NT and after NT were both identified as independent risk factors for overall survival (OS) and disease-free survival (DFS). The combination of low VAT and GLIM-defined malnutrition showed a higher hazard ratio for the prediction of OS and DFS both before NT and after NT. Conclusions: The addition of VAT assessment using CT improved the predictive value of GLIM-defined malnutrition for NT-related AEs and long-term survival in GC patients treated with NT followed by radical gastrectomy, which further supports the prognostic importance of assessing adipose tissue simultaneously during the routine nutritional assessment in patients with cancer.

## 1. Introduction

Gastric cancer (GC) is the fifth most common malignancy and the fourth leading cause of cancer-related death worldwide. In China, GC remains a major health burden, with over 450,000 new cases and 350,000 deaths per year, accounting for approximately 48.6% of GC-related deaths worldwide [1]. The standard treatment for locally advanced GC is D2 gastrectomy combined with perioperative therapy [2]. Despite recent advances in diagnosis and treatment, the prognosis of GC remains poor, with a five-year survival rate of only 35.9% in China [3]. Hence, the optimal treatment strategies for GC patients are continuously evolving, and comprehensive multidisciplinary therapy is gaining increasing attention and application.

Malnutrition frequently develops in patients with advanced GC due to the tumor itself or the side effects of various anticancer therapies and could result in adverse clinical outcomes, including increased treatment-related adverse events (AEs), more postoperative complications, a longer length of hospital stay, reduced tolerance of oncologic therapy, and poorer long-term survival [4]. Early identification of malnutrition is of crucial importance because it facilitates timely nutritional interventions to prevent further nutritional deterioration and its consequence. Previously, there have been a variety of nutritional screening or assessment tools to determine the nutritional status of patients, but there is an absence of consensus on diagnostic criteria for malnutrition in clinical practice.

The Global Leadership Initiative on Malnutrition (GLIM) consensus criteria has been recently published for diagnosing malnutrition in adults in clinical settings, based on a two-step approach for malnutritional risk screening and diagnostic assessment. The three phenotypic criteria (non-volitional weight loss, low body mass index (BMI), and reduced muscle mass) and two etiologic criteria (reduced food intake or assimilation and disease burden/inflammatory condition) were proposed in the second step of the GLIM framework. The diagnosis of GLIM-defined malnutrition requires to fulfill one phenotypic criterion and one etiologic criterion [5]. Many studies have reported the effectiveness of this novel criteria in diagnosing malnutrition and predicting clinical outcomes in several oncology populations. GLIM-defined malnutrition showed significant predictive value for treatment-related AEs, postoperative complications, and long-term survival [6,7,8,9,10,11]. However, some of the phenotypic criteria of the GLIM criteria might require further refinement or adjustment, such as the optimal thresholds and combinations of parameters to comprehensively reflect malnutrition.

A growing number of studies have reported that the assessment of adipose tissue could provide vital prognostic information among patients with cancer. The content and distribution of adipose tissue have shown significant prognostic value for determining cancer-related outcomes. Our previous study found that although high visceral adipose tissue (VAT) leads to an increased risk of postoperative complications, there is a notably improved survival outcome in GC patients who underwent radical surgery after neoadjuvant treatment (NT) [12]. Moreover, patients with advanced GC also have a poor prognosis when they experience a marked loss of VAT during NT [13]. Some recent studies also support the conclusion that low VAT was an independent risk factor for worse long-term survival in GC patients [14,15]. However, the GLIM criteria only include muscle mass as a component and do not include adipose tissue assessment, which is different from Patient-Generated Subjective Global Assessment (PG-SGA), a well-validated and widely used nutrition assessment tool for oncology populations [16]. Of note, recently, two large-scale cohort studies showed that additional fat mass assessment enhanced the prognostic value of GLIM criteria in patients with cancer. Patients with concurrent low-fat mass and GLIM-defined malnutrition had a greater death hazard compared with the well-nourished group. Among the two multicenter cohort studies, fat mass was assessed using bioelectrical impedance analysis (BIA) or the triceps skinfold (TSF) thickness [10,11]. Computed tomography (CT) examinations were routinely performed in the diagnosis and treatment of tumors in clinical practice. It has been recommended by GLIM criteria and applied more extensively for body composition assessment in patients with cancer. Hence, given its accessibility and importance, we speculated that VAT assessment using CT could also provide additional prognostic value in patients with cancer.

As far as we know, no previous study has investigated the relationship between GLIM-defined malnutrition and clinical outcomes in GC patients who underwent surgery after NT. The validity of the GLIM criteria has not been well-established in such patients. Therefore, in this present study, we aim to explore the prognostic value of GLIM-defined malnutrition before NT and after NT in GC patients treated with NT followed by radical gastrectomy and to investigate whether additional VAT assessment using CT could improve the predictive power of the GLIM criteria for NT-related AEs and long-term survival.

## 2. Materials and Methods

### 2.1. Patients

This retrospective study was conducted in a cohort of GC patients who underwent surgery after NT at the Department of General Surgery, Peking Union Medical College Hospital, from June 2016 to June 2020. The inclusion criteria were patients who: (1) were diagnosed with gastric adenocarcinoma by endoscopic biopsy; (2) received neoadjuvant chemotherapy (NAC) or neoadjuvant chemoradiotherapy (NCRT) before surgery; (3) underwent radical gastrectomy and D2 lymphadenectomy; (4) received abdominal CT examinations performed in our hospital before NT and after NT; (5) had pathologic response assessment using surgical specimens; (6) had complete clinicopathological data and follow-up data; (7) were aged 18–80 years. Exclusion criteria were patients who: (1) had the presence of distant metastasis; (2) received previous chemotherapy or radiotherapy at an outside institution; (3) were diagnosed with gastric stump cancer; (4) had other malignant tumors within five years before treatment; (5) had severe cardiovascular and cerebrovascular diseases, liver and kidney dysfunction, and other complications; (6) were pregnant and lactating women.

Indications of NT in our center are resectable locally advanced GC with clinical stage cT2-4N0-3M0. For tumor staging, abdominal CT, endoscopic ultrasonography, and abdominal magnetic resonance imaging examinations were routinely performed before NT and after NT [17]. Patients underwent one of the following three types of NAC regimens: (1) SOX chemotherapy consisting of oxaliplatin and S-1, (2) XELOX chemotherapy consisting of oxaliplatin and capecitabine, or (3) FOLFOX chemotherapy consisting of 5-FU, oxaliplatin, and leucovorin. Some patients with gastroesophageal junction cancer also received radiotherapy. Radiotherapy was administrated by intensity-modulated radiation therapy using 6-MV photons at 45 Gy given as 25 fractions of 1.8 Gy (5 fractions/week). All patients enrolled in this study underwent laparoscopic radical gastrectomy with D2 lymph node dissection. The research protocol was approved by the Institutional Review Board of Peking Union Medical College Hospital.

### 2.2. Data Acquisition

The following data of patients were acquired from the electronic medical record system: (1) baseline information, including age, sex, height, body weight, BMI, Charlson comorbidity index (CCI), the Nutritional Risk Screening 2002 (NRS2002), neutrophil-lymphocyte ratio (NLR), serum prealbumin (serum PA), and clinical stage; (2) NT details, including NT scheme and NAC regimen; (3) operative documents, including the type of resection and type of reconstruction; (4) tumor characteristics, including tumor location, post-neoadjuvant therapy stage (ypstage), pathological response to NT (tumor regression grading (TRG) systems of College of American Pathologists (CAP), CAP-TRG); (5) NT-related AEs, which were evaluated and classified according to Common Terminology Criteria for Adverse Events, version 4.0 [18]. Additionally, BMI was categorized into four groups: underweight (<18.5 kg/m^2^), normal (18.5–22.9 kg/m^2^), upper normal (23.0–24.9 kg/m^2^), overweight and obese (≥25.0 kg/m^2^) [19,20]. The NLR was used as an index of systemic inflammation and dichotomized at ≥3 [21]. A concentration of serum PA < 200 mg/L was considered low nutritional status [22]. Tumor stages, including the clinical stage and the ypstage, were carried out by the American Joint Committee on Cancer (AJCC) tumor staging (8th edition) [23]. All resected surgical specimens were examined by pathologists, and the pathological response to NT was evaluated according to the criteria of the CAP. According to the CAP-TRG system, patients were classified with complete (CAP 0), moderate (CAP 1), minimal (CAP 2), and poor (CAP 3) pathological responses [24].

### 2.3. Follow Up

All GC patients at our institution were required to have routine follow-up visits after surgery. The patients were followed via outpatient visits or telephone interviews until death, last contact, or May 2022. The overall survival (OS) was calculated from the date of surgical operation to the date of death (all-cause mortality) or the date of the last follow-up. The disease-free survival (DFS) was defined as the time from surgical operation to the time when cancer recurrence happened, death occurred, or the last follow-up.

### 2.4. Measurements of Body Composition

All GC patients enrolled in this study underwent abdominal CT within 1 month before NT and within 1 month before surgery in our institution. CT-based measurement is considered the gold standard for assessing body composition [25]. The cross-sectional areas of skeletal muscle and adipose tissue, including total skeletal muscle (TSM) and VAT, were measured on axial portal phase CT images at the level of third lumbar vertebra (L3) using ImageJ software. Tissue-specific attenuation values (Hounsfield unit, HU) were used to differentiate body composition, −29 to 150 HU for TSM and −150 to −50 HU for VAT [26].

The skeletal muscle index (SMI, cm^2^/m^2^) was calculated using the L3-TSM (cm^2^) normalized for height squared (m^2^). The sex-specific cut-off values for SMI were 52.4 cm^2^/m^2^ for men and 38.5 cm^2^/m^2^ for women, which is the most commonly used definition for prognosis studies among patients with cancer [27]. The cut-off values for VAT were based on our previous study using the optimal stratification method, which was 120 cm^2^ before NT and 106 cm^2^ after NT in both sexes [12].

### 2.5. Diagnosis of Malnutrition Using the GLIM Criteria

According to the GLIM criteria, a two-step approach was used for the malnutrition diagnosis [5]. In our center, we systematically perform the nutritional assessment for each patient at hospital admission. Firstly, the nutritional risk screening was performed for all patients within 24 h after admission using NRS2002. Then, for patients at risk of malnutrition (NRS2002 scores ≥ 3), at least one phenotypic criterion and one etiologic criterion should be present to diagnose GLIM-defined malnutrition. For the phenotypic criteria, the non-volitional weight loss (5–10% within the past 6 months, or >10% beyond 6 months), low BMI (<18.5 kg/m^2^ if <70 years, or <20 kg/m^2^ if >70 years), and reduced muscle mass (based on SMI derived from abdominal CT, <52.4 cm^2^/m^2^ for men or <38.5 cm^2^/m^2^ for women) were evaluated as described in the GLIM criteria. For the etiologic criteria, patients all fulfilled the disease burden-related etiologic criteria since all patients enrolled in this study were pathologically diagnosed with GC.

### 2.6. Statistical Analysis

The normality of continuous data was checked using the Shapiro–Wilk test. Categorical variables were presented as numbers (percentages). Continuous variables were presented as mean (standard deviation) if normally distributed or as median (interquartile range) if abnormally distributed and compared using the *t*-test and Mann–Whitney *U* test where appropriate. Survival curves were estimated using the Kaplan–Meier method and compared using the log-rank test. Univariate logistic analyses and Cox regression analyses were performed to identify potential risk factors for NT-related AEs, OS, and DFS. The univariate predictors for clinical outcomes with *p* < 0.15 were entered in a stepwise multivariate logistic or Cox regression model. Two separate multivariable analyses for NT-related AEs, OS, and DFS were performed, including one of the malnutrition diagnoses (GLIM-malnutrition or GLIM-malnutrition + low VAT), respectively. Only factors statistically significant in the final multivariate logistic or Cox regression model were shown. The odds ratio or hazard ratio, with a 95% confidence interval, was shown in the results. Two-sided *p* < 0.05 was regarded as statistically significant. All analyses were performed using the R software (version 4.1.3).

## 3. Results

### 3.1. Baseline Characteristics

A total of 182 GC patients who underwent radical gastrectomy after NT were included in this study, of which 135 (74.2%) patients were male and 47 (25.8%) patients were female, with a median age of 62 years. In the overall population, 172 (94.5%) patients received the SOX regimen, and 35 (19.2%) patients also underwent NCRT. Following NT, 29 (15.9%) patients achieved complete pathological response (CAP 0). Among the remaining 153 patients with CAP 1-3, 63 (34.7%) tumors were ypstage I, 45 (24.7%) were ypstage II, and 45 (24.7%) were ypstage III. The overall clinicopathological characteristics of the study population are shown in Table 1.

Based on the two-step approach in the GLIM criteria, it was found that 82 (45.1%) patients had malnutritional risk according to the NRS2002 assessment, and GLIM-defined malnutrition was subsequently identified in 66 (36.3%) patients before NT. Similarly, 66 (36.3%) patients were considered at malnutritional risk based on the NRS2002, and 55 (30.2%) patients had malnutrition assessed by the GLIM criteria after NT. Moreover, for the body composition, compared with those without malnutrition, GC patients with GLIM-defined malnutrition had lower TSM and VAT both before NT and after NT (Table 2). When low VAT was added to the diagnosis of malnutrition, 54 (29.7%) patients had additional low VAT before NT, and 39 (21.4%) patients had additional low VAT after NT in addition to GLIM-defined malnutrition.

### 3.2. NT-Related AEs

NT-related AEs of graded II or higher occurred in 53 (29.1%) of the GC patients during NT, including leukopenia or neutropenia (18, 9.9%), anemia (6, 3.3%), nausea or vomiting (27, 14.8%), diarrhea (6, 3.3%), stomatitis or rash (5, 2.7%), and liver function abnormalities (5, 2.7%). Univariate and multivariate analysis revealed that GLIM-defined malnutrition alone before NT was not associated with NT-related AEs (Table 3). However, the addition of low VAT to GLIM-defined malnutrition led to a significant predictive value for NT-related AEs in the univariate analysis. Concurrent low VAT and GLIM-defined malnutrition before NT remained an independent risk factor for overall NT-related AEs in multivariate logistic regression analysis (Table 3). Moreover, among these two malnutrition diagnoses, as shown in Table 4, the addition of low VAT to GLIM-defined malnutrition resulted in an improved specificity, accuracy, and positive predictive value for the prediction of NT-related AEs in GC patients.

### 3.3. Long-Term Outcomes

**Figure 1:**
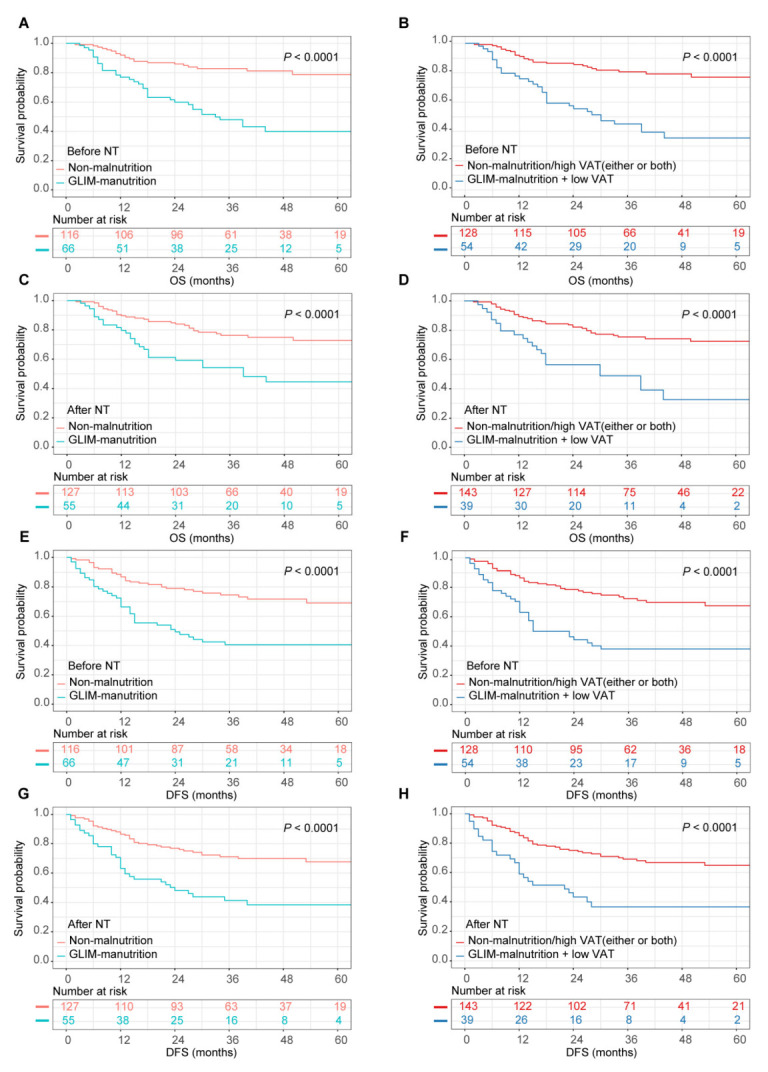
The associations between malnutrition diagnoses and long-term survival. GLIM, Global Leadership Initiative on Malnutrition; VAT, visceral adipose tissue; OS, overall survival; DFS, disease-free survival. For OS: (**A**) Kaplan–Meier curves stratified by GLIM-malnutrition before NT. (**B**) Kaplan–Meier curves stratified by GLIM-malnutrition plus low VAT before NT. (**C**) Kaplan–Meier curves stratified by GLIM-malnutrition after NT. (**D**) Kaplan–Meier curves stratified by GLIM-malnutrition plus low VAT after NT. For DFS: (**E**) Kaplan–Meier curves stratified by GLIM-malnutrition before NT. (**F**) Kaplan–Meier curves stratified by GLIM-malnutrition plus low VAT before NT. (**G**) Kaplan–Meier curves stratified by GLIM-malnutrition after NT. (**H**) Kaplan–Meier curves stratified by GLIM-malnutrition plus low VAT after NT.

During a median follow-up time of 43 months, 69 (37.9%) of 182 GC patients experienced cancer recurrence, and 58 (31.9%) patients died. Kaplan–Meier curves demonstrated that patients with GLIM-defined malnutrition before NT and after NT both had significantly worse OS and DFS than those in the non-malnutrition groups (Figure 1, all *p* < 0.05). Consistently, GLIM-defined malnutrition before NT and after NT were both identified as independent risk factors for OS and DFS in multivariate Cox regression analyses (Table 5 and Table 6).

Furthermore, in the Kaplan–Meier analyses, patients with concurrent low VAT and GLIM-defined malnutrition before NT and after NT both exhibited a higher risk of death and recurrence (Figure 1, all *p* < 0.05). Multivariate Cox analyses revealed that the combination of low VAT with GLIM-defined malnutrition led to a higher hazard ratio for the prediction of OS and DFS compared with GLIM-defined malnutrition alone both before NT and after NT (before NT, univariate analyses: 3.644 vs. 3.555 for OS, 2.823 vs. 2.749 for DFS; multivariate analyses: 2.963 vs. 2.635 for OS, 2.312 vs. 2.038 for DFS. After NT, univariate analyses: 2.989 vs. 2.682 for OS, 2.695 vs. 2.587 for DFS; multivariate analyses: 1.921 vs. 1.736 for OS, 1.701 vs. 1.662 for DFS) (Table 5 and Table 6).

## 4. Discussion

To the best of our knowledge, this is the first study evaluating GLIM-defined malnutrition before NT and after NT and its prognostic value on clinical outcomes in GC patients treated with NT, followed by radical gastrectomy. The prevalence of GLIM-defined malnutrition was 36.3% and 30.2% in GC patients before NT and after NT, respectively. Furthermore, we have presented evidence suggesting that additional VAT assessment using CT improved the predictive value of GLIM criteria for NT-related AEs and long-term survival. This suggests that VAT assessment can provide significant prognostic information in GC patients who underwent radical surgery after NT and further supports the prognostic importance of assessing adipose tissue simultaneously during the routine nutritional assessment in patients with cancer.

As a new consensus definition of malnutrition, the GLIM criteria have been initially validated in several oncology populations. Its value in predicting treatment-related AEs, postoperative complications, and long-term survival has also been described in various studies [6,7,8,9,10,11]. Multiple previous studies by Huang et al. showed that the GLIM criteria could be useful in predicting OS and DFS after radical gastrectomy for GC [7,8,9]. Consistent with the literature, this present study confirmed the significant predictive value of GLIM-defined malnutrition for OS and DFS both before NT and after NT in GC patients. Furthermore, the addition of low VAT before NT and after NT both increased the hazard ratio for the prediction of OS and DFS in GC patients treated with NT, followed by radical gastrectomy. Two recent large-scale multicenter studies also demonstrated that concurrent low-fat mass and GLIM-defined malnutrition were associated with greater death hazards in patients with cancer [10,11]. On the other hand, in terms of treatment-related AEs, a recent study demonstrated that patients with GLIM-defined malnutrition at admission had an increased risk of radiotherapy-induced severe toxicities and radiotherapy interruption [6]. However, in this study, we found no significant association between GLIM-defined malnutrition alone before NT and NT-related AEs in GC patients. This suggested that GLIM criteria may not be an optimal assessment tool for predicting NT-related AEs in GC patients during NT. Of note, the addition of low VAT to GLIM-malnutrition could improve the recognition of NT-related AEs in this cohort. It suggested that VAT assessment added supportive value to GLIM-defined malnutrition for the prediction of NT-related AEs in GC patients. From these results, the combination of low VAT and GLIM-defined malnutrition showed greater prognostic value than GLIM-defined malnutrition alone. Therefore, although it is not listed as a phenotypic criterion in the GLIM criteria, the addition of VAT assessment did significantly increase the performance of the GLIM criteria for diagnosing malnutrition.

It is interesting to note that additional VAT assessment using CT enhances the prognostic value of GLIM criteria in patients with GC who underwent radical surgery after NT in the present study. The impact of body composition on oncology outcomes has recently attracted great clinical interest. As is well known, CT-based measurement can differentiate body composition and is considered the gold standard for assessing skeletal muscle and adipose tissue mass [23]. Reduced muscle mass is one of the three phenotypic components with strong evidence to support its inclusion in the GLIM criteria, which provides more detailed and accurate information about body composition [5,28]. However, multiple prior studies have also noted the importance of VAT assessment and its impact on clinical outcomes in several oncology populations [12,13,14,15,29,30,31]. Assessment of adipose tissue has also been incorporated into many other well-validated nutritional assessment tools like PG-SGA [16]. In recent years, increasing evidence has shown that low VAT was associated with worse survival in patients with GC. As mentioned in our previous study, GC patients with low VAT before NT and after NT both had poorer long-term survival [12]. Likewise, recent findings from a larger retrospective cohort study suggested that low preoperative VAT mass was correlated to poorer prognosis after gastrectomy in advanced GC patients [14]. Another recent retrospective cohort study demonstrated that preoperative low VAT mass was an independent risk factor for poor compliance with adjuvant chemotherapy and an adverse prognostic factor for relapse-free survival (RFS) in patients with advanced GC who received adjuvant chemotherapy after radical gastrectomy [15]. Thus, the assessment of VAT is a key component of patient risk stratification, which may be underestimated when diagnosing malnutrition by the existing GLIM criteria. These findings could support including adipose tissue assessment as a component in GLIM criteria, at least for GC patients.

It is worth discussing these interesting associations revealed by the results of this present study. Undoubtedly, skeletal muscle and adipose tissue mass both reflect nutritional status and energy reserves. More importantly, adipose tissue loss often occurs more rapidly and earlier than muscle loss in progressive cancer cachexia, which could explain why patients with decreased VAT or marked loss of VAT have a less favorable prognosis [32]. Additionally, as a critical and active multifunctional organ, VAT secretes a variety of adipokines and cytokines that are involved in immunologic, metabolic, and endocrine activity [33]. On the other hand, a strong relationship between low VAT and low stromal lymphocyte infiltration has been reported in a large cohort study of patients with nasopharyngeal cancer [30]. It is proposed that VAT adipocytes could support immune activities by providing necessary unsaturated fatty acids [34]. This evidence may explain the protective effects and prognostic value of VAT on cancer.

Several limitations of this study should be noted. Firstly, this was a single-center, retrospective cohort study. Thus, due to the limited sample size, these patients were not further stratified into combination GLIM-malnutrition with high VAT, combination GLIM-malnutrition with low VAT, and well-nourished groups for more comprehensive analyses and gaining additional insights. More sufficient and powerful evidence about the additional prognostic value of VAT assessment to GLIM-defined malnutrition would be provided if these analyses were performed. Secondly, the cut-off value of VAT has not been well-established in the different populations. Hence, we used the cut-off values of VAT before NT and after NT identified in our previous study. Furthermore, due to the retrospective nature of this study, data on other supportive measures recommended by GLIM consensus, like muscle function, were not collected. Detailed information - about nutritional support, such as oral nutrition supplementation (ONS), were not prospectively collected. Additionally, other known prognostic markers of clinical outcomes in GC, such as human epidermal growth factor receptor 2 (HER2) expression levels and Lauren classification, were also not incorporated into the analyses. Hence, to address the above issues, well-designed prospective multicenter studies are required to further validate our findings.

## 5. Conclusions

In conclusion, the present study revealed that the addition of VAT assessment using CT enhanced the prognostic value of GLIM criteria for the prediction of NT-related AEs and long-term survival in patients with GC who underwent radical gastrectomy after NT. Due to its importance, adipose tissue assessment should be incorporated into routine nutritional assessment protocols in GC patients. More detailed nutrition evaluation and individualized nutritional interventions are needed to optimize strategies and improve clinical outcomes in clinical practice.

## Figures and Tables

**Table 1:** Patient characteristics, neoadjuvant treatments, operative details, and tumor characteristics.

Characteristics		Overall Cohort (*n* = 182)
*Patient characteristics*		
Age, y		62 (15.0)
Sex		
	Male	135 (74.2)
	Female	47 (25.8)
Height, m		1.7 (0.1)
CCI		
	2	122 (67.0)
	≥3	60 (33.0)
Clinical stage		
	II	46 (25.3)
	III–IV	136 (74.7)
Before NT		
Body weight, kg		67.4 (10.6)
BMI, kg/m^2^		
	<18.5	7 (3.8)
	18.5–22.9	71 (39.0)
	23–24.9	45 (24.8)
	≥25	59 (32.4)
NRS2002		
	<3	100 (54.9)
	≥3	82 (45.1)
NLR		
	≤3	144 (79.1)
	>3	38 (20.9)
Serum PA, mg/L		
	<200	46 (25.3)
	≥200	136 (74.7)
After NT		
Body weight, kg		66 (15.3)
BMI, kg/m^2^		
	<18.5	8 (4.4)
	18.5–22.9	71 (39.0)
	23–24.9	44 (24.2)
	≥25	59 (32.4)
NRS2002		
	<3	116 (63.7)
	≥3	66 (36.3)
NLR		
	≤3	149 (81.9)
	>3	33 (18.1)
Serum PA, mg/L		
	<200	43 (23.6)
	≥200	139 (76.4)
*Neoadjuvant treatments*		
NT scheme		
	NAC	147 (80.8)
	NCRT	35 (19.2)
NAC regimen		
	SOX	172 (94.5)
	XELOX/FOLFOX	10 (5.5)
*Operations performed*		
Type of resection		
	Total gastrectomy	87 (47.8)
	Distal gastrectomy	91 (50.0)
	Proximal gastrectomy	4 (2.2)
Type of reconstruction		
	Roux-en-Y	106 (58.2)
	Billroth I	76 (41.8)
*Tumor characteristics*		
Tumor location		
	Upper	42 (23.1)
	Middle	46 (25.3)
	Lower	94 (51.6)
ypstage		
	0	29 (15.9)
	I	63 (34.7)
	II	45 (24.7)
	III	45 (24.7)
CAP-TRG		
	0	29 (15.9)
	1	19 (10.4)
	2	70 (38.5)
	3	64 (35.2)

Abbreviations: CCI, Charlson comorbidity index; NT, neoadjuvant treatment; BMI, body mass index; NRS2002, the Nutritional Risk Screening 2002; NLR, Neutrophil-lymphocyte ratio; Serum PA, Serum prealbumin; NAC, neoadjuvant chemotherapy; NCRT, neoadjuvant chemoradiotherapy; ypstage, post-neoadjuvant therapy stage; CAP, College of American Pathologists; TRG, tumor regression grading. **-** Values for continuous variables are given as mean (standard deviation) or median (interquartile range). Categorical variables are expressed as numbers (percentages).

**Table 2:** Association between body composition and GLIM-defined malnutrition.

	Before NT			After NT		
	Non-Malnutrition (*n* = 116)	GLIM-Malnutrition (*n* = 66)	*p* Value	Non-Malnutrition (*n* = 127)	GLIM-Malnutrition (*n* = 55)	*p* Value
TSM (cm^2^)	144.6 ± 49.7	128.8 ± 25.4	**0.001**	149.0 ± 51.4	122.0 ± 16.8	**<0.001**
VAT (cm^2^)	140.3 ± 69.0	75.8 ± 67.0	**<0.001**	108.8 ± 103.7	71.8 ± 78.8	**0.001**

Abbreviations: TSM, total skeletal muscle; VAT, visceral adipose tissue; NT, neoadjuvant treatment; GLIM, the Global Leadership Initiative on Malnutrition. Values for continuous variables are given as mean ± standard deviation or median ± interquartile range. The bold values represent statistical significance, *p* < 0.05.

**Table 3:** Univariate and multivariate analysis for NT-related adverse events.

	Univariable Analysis		Multivariate Analysis			
Variables			GLIM-Malnutrition		GLIM-Malnutrition + Low VAT	
	OR (95% CI)	*p* Value	OR (95% CI)	*p* Value	OR (95% CI)	*p* Value
Age (≥65)	0.881 (0.446–1.742)	0.717				
Sex (female)	2.952 (1.467–5.941)	**0.002**	3.232 (1.576–6.626)	**0.001**	3.198 (1.539–6.645)	**0.002**
CCI (≥3)	0.428 (0.201–0.908)	**0.027**				
NT regimen (NCRT)	2.151 (1.002–4.621)	0.05	2.481 (1.119–5.503)	**0.025**	2.651 (1.172–5.996)	**0.019**
Clinical stage (III-IV/II)	0.919 (0.443–1.907)	0.821				
Before NT						
BMI (≥23)	0.781 (0.410–1.486)	0.452				
NLR (>3)	1.159 (0.535–2.514)	0.708				
Serum PA (<200)	1.855 (0.916–3.758)	0.086				
GLIM-malnutrition	1.714 (0.891–3.298)	0.106				
GLIM-malnutrition + low VAT	2.151 (1.093–4.233)	**0.027**			2.234 (1.095–4.558)	**0.027**

Abbreviations: NT, neoadjuvant treatment; GLIM, the Global Leadership Initiative on Malnutrition; VAT, visceral adipose tissue; CCI, Charlson comorbidity index; NCRT, neoadjuvant chemoradiotherapy; BMI, body mass index; NLR, Neutrophil-lymphocyte ratio; Serum PA, Serum prealbumin; OR, odds ratio; CI, confidence interval. Note: One of the malnutrition diagnoses (GLIM-malnutrition or GLIM-malnutrition + low VAT) was incorporated into Multivariate analysis. **-** The bold values represent statistical significance, *p* < 0.05.

**Table 4:** Sensitivity and specificity of different malnutrition diagnoses for the prediction of NT-related adverse events.

Factors	Sensitivity (%)	Specificity (%)	Accuracy (%)	PPV (%)	NPV (%)
GLIM-malnutrition	45.28	67.44	60.99	36.36	75.00
GLIM-malnutrition + low VAT	41.51	75.19	65.38	40.74	75.78

Abbreviations: NT, neoadjuvant treatment; GLIM, the Global Leadership Initiative on Malnutrition; VAT, visceral adipose tissue; PPV, positive predictive value; NPV, negative predictive value.

**Table 5:** Univariate and multivariate analysis for prognostic factors for overall survival.

	Univariable Analysis		Multivariate Analysis							
			Before NT				After NT			
Variables			GLIM-Malnutrition		GLIM-Malnutrition + Low VAT		GLIM-Malnutrition		GLIM-Malnutrition + Low VAT	
	HR (95% CI)	*p* Value	HR (95% CI)	*p* Value	HR (95% CI)	*p* Value	HR (95% CI)	*p* Value	HR (95% CI)	*p* Value
Age (≥65)	1.153 (0.675–1.971)	0.602								
Sex (female)	1.325 (0.759–2.312)	0.322								
CCI (≥3)	0.811 (0.455–1.443)	0.475								
NT regimen (NCRT)	0.960 (0.497–1.854)	0.903								
ypstage (III/0-II)	6.089 (3.580–10.357)	**<0.001**	4.931 (2.862–8.495)	**<0.001**	5.253 (3.065–9.003)	**<0.001**	5.194 (2.980–9.053)	**<0.001**	5.188 (2.986–9.015)	**<0.001**
Pathologic response (CAP 3/0-2)	2.184 (1.302–3.663)	**0.003**								
Before NT										
BMI (≥23)	0.630 (0.376–1.056)	0.08								
NLR (>3)	2.335 (1.365–3.992)	**0.002**								
Serum PA (<200)	1.372 (0.785–2.399)	0.267								
GLIM-malnutrition	3.555 (2.089–6.051)	**<0.001**	2.635 (1.527–4.547)	**0.001**						
GLIM-malnutrition + low VAT	3.644 (2.168–6.123)	**<0.001**			2.963 (1.750–5.016)	**<0.001**				
After NT										
BMI (≥23)	0.788 (0.471–1.319)	0.365								
NLR (>3)	0.818 (0.401–1.668)	0.581								
Serum PA (<200)	2.266 (1.324–3.880)	**0.003**								
GLIM-malnutrition	2.682 (1.600–4.494)	**<0.001**					1.736 (1.010–2.985)	**0.046**		
GLIM-malnutrition + low VAT	2.989 (1.752–5.102)	**<0.001**							1.921 (1.101–3.352)	**0.022**

**Table 6:** Univariate and multivariate analysis for prognostic factors for disease-free survival.

	Univariable Analysis		Multivariate Analysis							
			Before NT				After NT			
Variables			GLIM-Malnutrition		GLIM-Malnutrition + Low VAT		GLIM-Malnutrition		GLIM-Malnutrition + Low VAT	
	HR (95% CI)	*p* Value	HR (95% CI)	*p* Value	HR (95% CI)	*p* Value	HR (95% CI)	*p* Value	HR (95% CI)	*p* Value
Age (≥65)	1.132 (0.693–1.850)	0.62								
Sex (female)	1.298 (0.777–2.168)	0.319								
CCI (≥3)	0.955 (0.576–1.585)	0.859								
NT regimen (NCRT)	1.197 (0.675–2.122)	0.538								
ypstage (III/0-II)	6.249 (3.846–10.152)	**<0.001**	5.394 (3.281–8.868)	**<0.001**	5.647 (3.452–9.239)	**<0.001**	5.394 (3.245–8.967)	**<0.001**	5.481 (3.309–9.081)	**<0.001**
Pathologic response (CAP 3/0-2)	2.335 (1.454–3.752)	**<0.001**								
Before NT										
BMI (≥23)	0.638 (0.398–1.024)	0.063								
NLR (>3)	2.140 (1.296–3.532)	**0.003**								
Serum PA (<200)	1.486 (0.895–2.465)	0.126								
GLIM-malnutrition	2.749 (1.709–4.423)	**<0.001**	2.038 (1.252–3.319)	**0.004**						
GLIM-malnutrition + low VAT	2.823 (1.757–4.536)	**<0.001**			2.312 (1.429–3.741)	**0.001**				
After NT										
BMI (≥23)	0.890 (0.554–1.431)	0.631								
NLR (>3)	1.206 (0.670–2.170)	0.532								
Serum PA (<200)	2.195 (1.336–3.607)	**0.002**								
GLIM-malnutrition	2.587 (1.609–4.159)	**<0.001**					1.662 (1.009–2.736)	**0.046**		
GLIM-malnutrition + low VAT	2.695 (1.637–4.437)	**<0.001**							1.701 (1.012–2.859)	**0.045**

Abbreviations: NT, neoadjuvant treatment; GLIM, the Global Leadership Initiative on Malnutrition; VAT, visceral adipose tissue; CCI, Charlson comorbidity index; NCRT, neoadjuvant chemoradiotherapy; ypstage, post-neoadjuvant therapy stage; CAP, tumor regression grading of College of American Pathologists; BMI, body mass index; NLR, Neutrophil-lymphocyte ratio; Serum PA, Serum prealbumin; HR, hazard ratio; CI, confidence interval. Note: One of the malnutrition diagnoses (GLIM-malnutrition or GLIM-malnutrition + low VAT) was incorporated into Multivariate analysis. **-** The bold values represent statistical significance, *p* < 0.05.

## Data Availability

The original data supporting the conclusion of this paper will be provided by the author without improper retention.

## References

[1] Sung H., Ferlay J., Siegel R.L., Laversanne M., Soerjomataram I., Jemal A., Bray F. (2021). Global Cancer Statistics 2020: GLOBOCAN Estimates of Incidence and Mortality Worldwide for 36 Cancers in 185 Countries. CA Cancer J. Clin..

[2] Zhang X., Liang H., Li Z., Xue Y., Wang Y., Zhou Z., Yu J., Bu Z., Chen L., Du Y. (2021). Perioperative or postoperative adjuvant oxaliplatin with S-1 versus adjuvant oxaliplatin with capecitabine in patients with locally advanced gastric or gastro-oesophageal junction adenocarcinoma undergoing D2 gastrectomy (RESOLVE): An open-label, superiority and non-inferiority, phase 3 randomised controlled trial. Lancet Oncol..

[3] Allemani C., Matsuda T., Di Carlo V., Harewood R., Matz M., Niksic M., Bonaventure A., Valkov M., Johnson C.J., Esteve J. (2018). Global surveillance of trends in cancer survival 2000-14 (CONCORD-3): Analysis of individual records for 37 513 025 patients diagnosed with one of 18 cancers from 322 population-based registries in 71 countries. Lancet.

[4] Arends J., Bachmann P., Baracos V., Barthelemy N., Bertz H., Bozzetti F., Fearon K., Hutterer E., Isenring E., Kaasa S. (2017). ESPEN guidelines on nutrition in cancer patients. Clin. Nutr..

[5] Cederholm T., Jensen G.L., Correia M., Gonzalez M.C., Fukushima R., Higashiguchi T., Baptista G., Barazzoni R., Blaauw R., Coats A. (2019). GLIM criteria for the diagnosis of malnutrition—A consensus report from the global clinical nutrition community. Clin. Nutr..

[6] Zhang Z., Wan Z., Zhu Y., Wan H. (2022). Predictive validity of the GLIM criteria in treatment outcomes in cancer patients with radiotherapy. Clin. Nutr..

[7] Huang D.D., Wu G.F., Luo X., Song H.N., Wang W.B., Liu N.X., Yu Z., Dong Q.T., Chen X.L., Yan J.Y. (2021). Value of muscle quality, strength and gait speed in supporting the predictive power of GLIM-defined malnutrition for postoperative outcomes in overweight patients with gastric cancer. Clin. Nutr..

[8] Huang D.D., Yu D.Y., Wang W.B., Song H.N., Luo X., Wu G.F., Chen X.L., Yu Z., Yan J.Y. (2022). Global leadership initiative in malnutrition (GLIM) criteria using hand-grip strength adequately predicts postoperative complications and long-term survival in patients underwent radical gastrectomy for gastric cancer. Eur. J. Clin. Nutr..

[9] Huang D.D., Yu D.Y., Song H.N., Wang W.B., Luo X., Wu G.F., Yu Z., Liu N.X., Dong Q.T., Chen X.L. (2021). The relationship between the GLIM-defined malnutrition, body composition and functional parameters, and clinical outcomes in elderly patients undergoing radical gastrectomy for gastric cancer. Eur. J. Surg. Oncol..

[10] Yin L., Song C., Cui J., Wang N., Fan Y., Lin X., Zhang L., Zhang M., Wang C., Liang T. (2022). Low fat mass index outperforms handgrip weakness and GLIM-defined malnutrition in predicting cancer survival: Derivation of cutoff values and joint analysis in an observational cohort. Clin. Nutr..

[11] Yin L., Fan Y., Lin X., Zhang L., Li N., Liu J., Guo J., Zhang M., He X., Liu L. (2022). Fat mass assessment using the triceps skinfold thickness enhances the prognostic value of the Global Leadership Initiative on Malnutrition criteria in patients with lung cancer. Br. J. Nutr..

[12] Zhang Y., Li Z., Jiang L., Xue Z., Ma Z., Kang W., Ye X., Liu Y., Jin Z., Yu J. (2021). Impact of body composition on clinical outcomes in people with gastric cancer undergoing radical gastrectomy after neoadjuvant treatment. Nutrition.

[13] Zhang Y., Li Z., Jiang L., Xue Z., Ma Z., Kang W., Ye X., Liu Y., Jin Z., Yu J. (2021). Marked loss of adipose tissue during neoadjuvant therapy as a predictor for poor prognosis in patients with gastric cancer: A retrospective cohort study. J. Hum. Nutr. Diet..

[14] Matsui R., Inaki N., Tsuji T. (2022). Impact of visceral adipose tissue on long-term outcomes after gastrectomy for advanced gastric cancer. Nutrition.

[15] Matsui R., Inaki N., Tsuji T. (2021). Impact of visceral adipose tissue on compliance of adjuvant chemotherapy and relapse-free survival after gastrectomy for gastric cancer: A propensity score matching analysis. Clin. Nutr..

[16] Ottery F.D. (1996). Definition of standardized nutritional assessment and interventional pathways in oncology. Nutrition.

[17] Zhang Y., Yu J. (2020). The role of MRI in the diagnosis and treatment of gastric cancer. Diagn. Interv. Radiol..

[18] Institute N.C. (2009). Common Terminology Criteria for Adverse Events (CTCAE), version 4.0.

[19] WHO (2000). The Asia-Pacific Perspective: Redefining Obesity and Its Treatment.

[20] Teufel F., Seiglie J.A., Geldsetzer P., Theilmann M., Marcus M.E., Ebert C., Arboleda W.A.L., Agoudavi K., Andall-Brereton G., Aryal K.K. (2021). Body-mass index and diabetes risk in 57 low-income and middle-income countries: A cross-sectional study of nationally representative, individual-level data in 685 616 adults. Lancet.

[21] Feliciano E.M.C., Kroenke C.H., Meyerhardt J.A., Prado C.M., Bradshaw P.T., Kwan M.L., Xiao J., Alexeeff S., Corley D., Weltzien E. (2017). Association of Systemic Inflammation and Sarcopenia with Survival in Nonmetastatic Colorectal Cancer: Results From the C SCANS Study. JAMA Oncol..

[22] Jia R.R., Zhong J.H., Huo R.R., Su Q.B., Xiang X., Zhao F.L., Qin Z.B., Chen J.H., Liao Y.Y., Ma L. (2019). Correlation between serum prealbumin and prognosis of patients with hepatocellular carcinoma after hepatectomy. J. Surg. Oncol..

[23] Amin M.B., Edge S.B., Greene F.L., Byrd D.R., Brookland R.K., Washington M.K., Gershenwald J.E., Compton C.C., Hess K.R., Sullivan D.C. (2017). AJCC Cancer Staging Manual.

[24] Burgart L.J., Chopp W.V., Jain D. (2020). Protocol for the Examination of Specimens from Patients with Carcinoma of the Stomach. https://documents.cap.org/protocols/Stomach_4.2.1.0.REL_CAPCP.pdf.

[25] Seidell J.C., Bakker C.J., van der Kooy K. (1990). Imaging techniques for measuring adipose-tissue distribution--a comparison between computed tomography and 1.5-T magnetic resonance. Am. J. Clin. Nutr..

[26] Gomez-Perez S.L., Haus J.M., Sheean P., Patel B., Mar W., Chaudhry V., McKeever L., Braunschweig C. (2016). Measuring Abdominal Circumference and Skeletal Muscle from a Single Cross-Sectional Computed Tomography Image: A Step-by-Step Guide for Clinicians Using National Institutes of Health ImageJ. JPEN J. Parenter. Enter. Nutr..

[27] Prado C.M., Lieffers J.R., McCargar L.J., Reiman T., Sawyer M.B., Martin L., Baracos V.E. (2008). Prevalence and clinical implications of sarcopenic obesity in patients with solid tumours of the respiratory and gastrointestinal tracts: A population-based study. Lancet Oncol..

[28] Barazzoni R., Jensen G.L., Correia M., Gonzalez M.C., Higashiguchi T., Shi H.P., Bischoff S.C., Boirie Y., Carrasco F., Cruz-Jentoft A. (2022). Guidance for assessment of the muscle mass phenotypic criterion for the Global Leadership Initiative on Malnutrition (GLIM) diagnosis of malnutrition. Clin. Nutr..

[29] Harada K., Baba Y., Ishimoto T., Kosumi K., Tokunaga R., Izumi D., Ida S., Imamura Y., Iwagami S., Miyamoto Y. (2015). Low Visceral Fat Content is Associated with Poor Prognosis in a Database of 507 Upper Gastrointestinal Cancers. Ann. Surg. Oncol..

[30] He W.Z., Jiang C., Liu L.L., Yin C.X., Rong Y.M., Hu W.M., Yang L., Wang L., Jin Y.N., Lin X.P. (2020). Association of body composition with survival and inflammatory responses in patients with non-metastatic nasopharyngeal cancer. Oral. Oncol..

[31] Kaneko G., Miyajima A., Yuge K., Yazawa S., Mizuno R., Kikuchi E., Jinzaki M., Oya M. (2015). Visceral obesity is associated with better recurrence-free survival after curative surgery for Japanese patients with localized clear cell renal cell carcinoma. Jpn. J. Clin. Oncol..

[32] Fouladiun M., Korner U., Bosaeus I., Daneryd P., Hyltander A., Lundholm K.G. (2005). Body composition and time course changes in regional distribution of fat and lean tissue in unselected cancer patients on palliative care--correlations with food intake, metabolism, exercise capacity, and hormones. Cancer.

[33] Doyle S.L., Donohoe C.L., Lysaght J., Reynolds J.V. (2012). Visceral obesity, metabolic syndrome, insulin resistance and cancer. Proc. Nutr. Soc..

[34] Mathis D. (2013). Immunological goings-on in visceral adipose tissue. Cell Metab..

